# Astrocytes Optimize the Synaptic Transmission of Information

**DOI:** 10.1371/journal.pcbi.1000088

**Published:** 2008-05-30

**Authors:** Suhita Nadkarni, Peter Jung, Herbert Levine

**Affiliations:** 1 Center of Theoretical Biological Physics, University of California San Diego, La Jolla, California, United States of America; 2 Department of Physics and Astronomy, Ohio University, Athens, Ohio, United States of America; UFR Biomédicale de l'Université René Descartes, France

## Abstract

Chemical synapses transmit information via the release of neurotransmitter-filled vesicles from the presynaptic terminal. Using computational modeling, we predict that the limited availability of neurotransmitter resources in combination with the spontaneous release of vesicles limits the maximum degree of enhancement of synaptic transmission. This gives rise to an optimal tuning that depends on the number of active zones. There is strong experimental evidence that astrocytes that enwrap synapses can modulate the probabilities of vesicle release through bidirectional signaling and hence regulate synaptic transmission. For low-fidelity hippocampal synapses, which typically have only one or two active zones, the predicted optimal values lie close to those determined by experimentally measured astrocytic feedback, suggesting that astrocytes optimize synaptic transmission of information.

## Author Summary

Release of chemical (neurotransmitter)-filled vesicles at neuronal junctions called synapses leads to transmission of information between neurons. In a successful synaptic transmission, a voltage spike (action potential) generated by a presynaptic neuron initiates neurotransmitter vesicle release and leads to a small current in the postsynaptic neuron. For many synapses in the central nervous system, the probability that a neurotransmitter vesicle is released in response to an action potential is conspicuously small, raising the question whether transmission failures can in any way prove advantageous. Apart from “induced vesicle release” (in response to an action potential), vesicles are also released asynchronously (in absence of an action potential). An induced release probability that is too small samples the information poorly, as many of the incoming action potentials do not result in a postsynaptic current response. Maximizing induced release in order to maximize information transmission at a synapse is accompanied by the exceptionable outcome of increased asynchronous release; in addition, both these releases draw from the same neurotransmitter resource pool. A large release rate thus comprising both induced as well as asynchronous release of vesicles can suppress synaptic transmission via either depletion of neurotransmitter resources or desensitization of postsynaptic receptors. In this paper, we propose that the competing dynamics of induced and asynchronous vesicle release gives rise to an optimal release probability. Further, by comparing experimental data of astrocyte-enhanced synaptic transmission with simulations, we argue that synapses enwrapped by astrocytes operate close to our predicted optimum. This optimality is achieved through a closed-loop control circuitry that involves the presynaptic neuron and the synaptic astrocyte.

## Introduction

Optimization principles for the nervous system have long been discussed on various levels of organization. Laughlin and Sejnowski [1] have argued that the brain evolved around design principles of optimizing energy consumption and conserving space, time and material resources for information processing. It has been proposed that the increase in wiring density needed to reduce energy consumption is constrained by an increase in channel noise, setting the minimum value of an axon diameter to be 0.1 µm [2],[3]. An optimization principle also holds true for the volume fraction (3/5) occupied by dendrites and axons in the grey matter so as to minimize conduction delays and passive cable attenuation and to maximize the density of synapses [4] in the nematode nervous system.

In this paper we propose a novel optimization principle for neurotransmitter release rates that operates at a lower level of organization, that of an individual synapse. At a synapse, vesicles of neurotransmitter are released, signaling the arrival of a presynaptic action potential to the postsynaptic neuron. The probabilities of vesicle release, however, are often conspicuously poor, ranging from 0.1 to 0.5 for hippocampal synapses [5]. This observation lends itself to the question of whether there is an optimality principle that would predict such small values of vesicle release as advantageous? In previous work [6], we introduced a preliminary modeling framework for considering the astrocyte-mediated increase in action potential induced release. Here, we use this framework and include for the first time the increase in asynchronous release; this increase is due to the prolonged elevation of presynaptic calcium concentration caused by astrocytic signaling. Additionally, we explicitly take into account the available neurotransmitter resource as a function of release rate with appropriate neurotransmitter recycling time constants; this is crucial because both types of release utilize neurotransmitter resource from the same vesicle pool. Our simulations predict that under the dual constraints of finite neurotransmitter resources and spontaneous release processes, a low transmission probability indeed optimizes the average information content of synaptic transmission. We further show that the well-established bidirectional signaling between neurons and synaptic astrocytes allows a low-fidelity synapse to find this optimal value.

Sustained stimuli can cause activity dependent depression of synaptic transmission via several pathways. Amongst them reduction in vesicular glutamate concentration (quantal size) and receptor desensitization play a significant role in lowering the amplitude of the postsynaptic response at central glutamatergic synapses [7]–[10]. Here we focus on the quantal size issue. Our hypothesis is outlined as follows: A large increase in the probability of vesicle release leads to depression of the postsynaptic response and hence reduced information transmission. Conversely, if the vesicle release probability is too small, many presynaptically arriving action potentials will not lead to any postsynaptic response, and hence information transmission will be poor as well. We suggest that the optimum in between those limits can be attained by a modulation of the resting presynaptic calcium level. This modulation is achieved in practice via bidirectional dynamic signaling between an astrocyte and the synapse.

The key regulator of vesicle release probability is presynaptic Ca^2+^, which can be modulated by neuronal activity dependent feedback from synaptic astrocytes. A single astrocyte can potentially oversee around 100 000 synaptic contacts and more than 50% of the excitatory synapses in the hippocampus are associated with astrocytic processes with varying degrees of coverage [11],[12]. This architecture strongly suggests that astrocytes participate in the dynamics of neuronal networks, and certainly, in the dynamics of a single synapse. This notion has led to the concept of a ‘tripartite synapse’ – a pre and a postsynaptic terminal modulated by an astrocyte [13].

**Figure 1:**
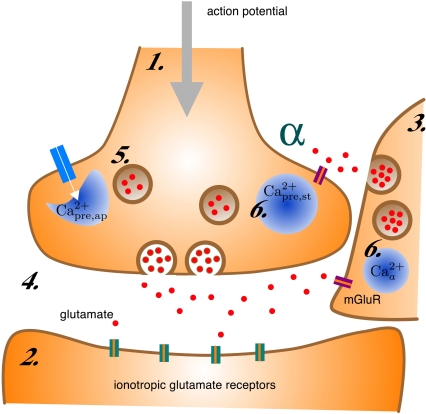
A schematic of synaptic transmission at a glutamatergic tripartite synapse. Arrival of an action potential opens voltage gated Ca^2+^ channels (white arrow), leading to a quick flux of calcium in the presynaptic terminal (1) that lasts between 1–2 ms. Glutamate (red circles) release in the synaptic cleft (4) takes place due to Ca^2+^ binding to vesicle release machinery and initiates a small inward current in the postsynaptic terminal (2) by activating the ionotropic receptors (green and orange bars). For hippocampal synapses, the probability of vesicle (5) release is small, averaging at approximately 0.2. Activation of metabotropic glutamate receptors (indigo and orange bars) on the adjacent astrocytic process (3) due to glutamate binding initiates release of Ca^2+^ from internal stores (6) in the astrocyte from IP_3_ R-mediated Ca^2+^ calcium channels causing an elevation in intracellular [Ca^2+^]. Intracellular [Ca^2+^]-dependent glutamate release from astrocytes triggers opening of Ca^2+^ stores (6) in the presynaptic terminal. Availability of two distinct sources of Ca^2+^ due to participation of the astrocyte increases neurotransmitter release rates. The astrocyte to neuron coupling parameter *α* governs the increase in presynaptic [Ca^2+^] and therefore the extent of potentiation.

Although astrocytes are not electrically excitable they can respond to local neuronal activity with an elevation in intracellular calcium concentration [14]. A characteristic calcium response in the astrocyte lasts for a few seconds, thousand times slower than an action potential. Intracellular calcium elevations in the astrocyte can trigger the release of a variety of gliotransmitters including glutamate. The exact biophysical mechanism underlying this release is uncertain; calcium dependent vesicular release of glutamate has been reported under physiological conditions [15], but several other mechanisms of release have also been reported [16],[17]. Because of this uncertainty, the kinetics of the glutamate release from astrocytes have not yet been quantified. The released glutamate from astrocytes can bind to presynaptic mGluRs [18],[19] and modulate synaptic transmission for several seconds, increasing spontaneous release rates and enhancing the probability of a vesicle release to stimulus [20] (see Figure 1). Activation of group I mGluRs (mGluR1 and mGluR5) are coupled to IP_3_ production and associated IP_3_ receptor channel mediated opening of Ca^2+^ stores [21]. An elevation in intracellular Ca^2+^ due to opening of these stores [22],[23] and the concomitant modulation in neurotransmitter release have been reported as a result of physiological activation of presynaptic mGluRs [24].

In our model we assume that glutamate released from the astrocytes that binds to presynaptic mGluRs gives rise to a sustained increase in intracellular Ca^2+^ concentration in the presynaptic terminal. Our methodology consists of combining a model for presynaptic vesicle release with a model for the astrocytic calcium dynamics to investigate the role of astrocyte-neuron interaction for selecting the vesicle release rate. To circumvent the limitation of data regarding the specific biophysical mechanism of glutamate release from astrocyte, we directly incorporate the experimentally determined change in neurotransmission as a result of astrocytic Ca^2+^ response to neuronal firing. This renders our model insensitive to the aforementioned uncertainty.

The increase in transmission probabilties as a result of astrocytic Ca^2+^ response to neuronal firing in hippocampal slices was investigated between GABAergic synapses by Kang et al. [20]. In our theoretical study, we assumed that their results continue to be valid for the case of excitatory glutamatergic synapses. More direct data on the increase in release probabilities of neurotransmitter between glutamatergic synapses mediated by the calcium response in the synaptic astrocyte has been recently reported by Perea et al. [19]. In accordance with our assumptions, this pathway of enhancing synaptic transmission was indeed characterized by activation of presynaptic mGluRs, by glutamate release from astrocytes. However, the original study by Kang et al. [20] is more immediately relevant to our modeling as there, presynaptic firing activity generates the calcium response in the astrocyte which in turn modulates transmission probability in what can be termed a closed-loop protocol. In contrast, an open-loop protocol is followed by Fiacco et al. and Perea et al. [18],[19], where external stimulation through caged Ca^2+^ and/or IP3 generates the calcium response in the astrocyte. In our model where we argue that the system operates at an optimal level, it becomes essential to consider a closed loop system. The underlying assumption is that Ca^2+^ sensor protein that allows for the release of a single unit of neurotransmitter follows the same general principles in central synapses [25] for both inhibitory and excitatory. Furthermore, the Ca^2+^ response of the astrocyte to neuronal firing rate in the model compares favorably with Pasti et al. [14], confirming the validity of our methodology.

## Results

We started with a model for a low-fidelity hippocampal synaptic junction [20] with two active zones and a baseline transmission probability of P≈0.2. Vesicle-release probabilities vary among hippocampal synapses and P≈0.2 is a value that has been reported as an average value [5] during the duration of one action potential. We used the Bertram model [26] (see Methods) to describe vesicle release, specifically for its dependence on the presynaptic Ca^2+^ concentration.

In addition to stimulated release during action potentials, spontaneous release of vesicles can also occur. The rate of action potential independent release also depends on the presynaptic Ca^2+^ concentration [27],[28] in a domain close to the release machinery. We phenomenologically modeled spontaneous release as a Markov process with experimentally determined rates. Of particular importance is the very sharp rise in the release rate with calcium; hence it is not possible to increase the fidelity of stimulated response without also increasing the spontaneous release. The spontaneous releases deplete neurotransmitter levels and lead to reduced evoked post-synaptic currents; they also prevent triggered release for a short refractory period [29] (∼6 msec,) immediately after each spontaneous event.

### There Is an Optimal Value of Presynaptic Calcium

**Figure 2:**
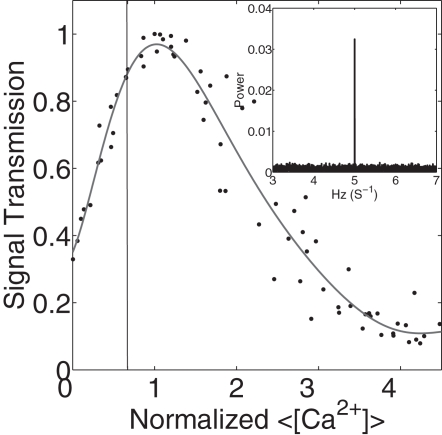
Synaptic signal transmission at a presynaptic action potential frequency of 5 Hz is shown as a function of the relative background calcium level. The calcium is normalized to the value that maximizes synaptic transmission. The vertical line denotes the value of calcium that yields the experimentally measured transmission probability of the tripartite synapse. The inset shows the power spectrum of the postsynaptic current events at the value of calcium that maximized the signal transmission.

We reasoned that the aforementioned competition between triggered and spontaneous events should give rise to an optimal value of the domain calcium concentration in the presynaptic terminal. In order to test this hypothesis, we simulated synaptic dynamics that arise in response to a periodic presynaptic train of action potentials with frequencies ranging from 5 Hz to 40 Hz at a fixed (non-evoked) presynaptic background calcium concentration (regardless of its origin). The transmission of the synapse was characterized by the transmission probability for a single spike and/or the power spectrum of the postsynaptic currents events. The power spectrum exhibits a sharp spike at the frequency of the presynaptic action potentials (see inset of Figure 2). The larger the amplitude of the peak, the more faithful is the postsynaptic current response, i.e. the more information has been transmitted through the synapse. In Figure 2, we display the amplitude of the peak in the power spectrum (which we term “synaptic transmission” from now on) as a function of the background calcium level (normalized to the value that maximizes “synaptic transmission”) at a frequency of 5 Hz. Transmission curves for similar frequencies are almost identical. For significantly larger frequencies of incoming spikes we find slightly larger transmitted information, which is due to short-term facilitation built into the Bertram model that we used for vesicle release.

**Figure 3:**
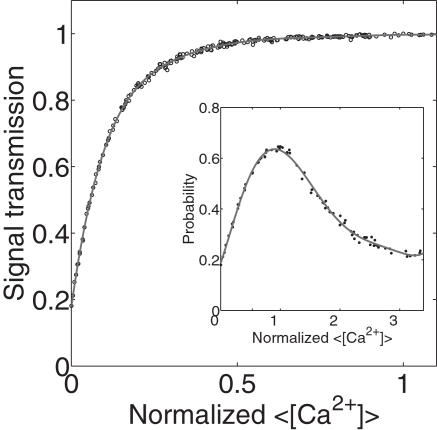
The signal transmission in the absence of spontaneous vesicle release events and single-spike transmission probability (inset) as a function of relative background calcium level. The vertical line (inset) denotes the value of calcium that yields the experimentally measured transmission probability of the tripartite synapse. The calcium is normalized to the value that maximizes the transmission probability.

We observed that synaptic transmission increases with relative Ca^2+^ until a maximum is reached. The peak value was essentially independent of frequency (data not shown). The decrease in synaptic transmission beyond its optimal value is due to the spontaneous release of vesicles, which becomes more frequent with increasing presynaptic Ca^2+^ concentration. To demonstrate this point, we simulated the synapse without the spontaneous release events. The results are shown in Figure 3. The synaptic transmission first increases with background calcium and then saturates; it does not exhibit a maximum. There are two mechanisms by which spontaneous release of vesicles can depress synaptic transmission: a) spontaneously released vesicles use up neurotransmitter resources and b) release sites are briefly inactivated after the release of a vesicle (see Methods). To see if the second effect is important, we plotted the single spike transmission probability, which is defined as the probability that a single presynaptic spike causes a postsynaptic current of any amplitude and is thus not directly related to what degree the vesicle is filled. The fact that the single-spike transmission probability, shown in the inset of Figure 3, also has a maximum albeit at a slightly shifted location, suggests that the latter mechanism is also important.

### Signaling to the Astrocyte Causes an Increase in the Single Spike Release Probability

**Figure 4:**
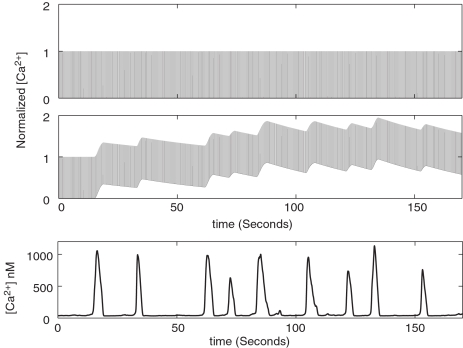
Presynaptic [Ca^2+^] in response to 20 Hz stimulus without (top panel) and with (center panel) feedback from the astrocyte. Opening of the voltage gated [Ca^2+^] channels due to an action potential gives rise to a [Ca^2+^] pulse that lasts 1.25 ms in our model. The *y*-axes have been normalized by [Ca^2+^] required to generate a release probability of P≈0.2 (300 µM for the Bertram, Sherman, and Stanley model [26]). (Bottom panel) Intracellular astrocytic [Ca^2+^] response in the astrocyte to 20 Hz of neuronal firing.

We extended our model to include the bidirectional signaling between the synapse and its enveloping astrocyte. This was done following the ideas originally discussed by Nadkarni and Jung [6]. Every time a vesicle is released, glutamate binds to astrocytic metabotropic receptors and initiates an IP_3_-dependent cascade leading to a rise in intracellular calcium (see Figure 4, bottom panel). Astrocytic Ca^2+^ response is oscillatory above a threshold IP_3_ concentration, as the Ca^2+^ flux through IP_3_Rs increases with increasing Ca^2+^. This positive feedback loop is called calcium induced calcium release (CICR), and is terminated due to slow inactivation of the IP_3_Rs at high Ca^2+^concentration. The typical width of the Ca^2+^ spike therefore is determined by the inactivation rate of the IP_3_Rs. The presence of a small number of IP_3_Rs renders the Ca^2+^ response stochastic [30] (See Methods, Equations 5–9). This stochasticity accounts for the “jitter” in the astrocytic calcium oscillations. Ca^2+^-dependent release of additional glutamate from the astrocyte activates neuronal metabotropic receptors and opens presynaptic calcium stores. This mechanism allows feedback from the astrocyte to regulate the vesicle release probability.

**Figure 5:**
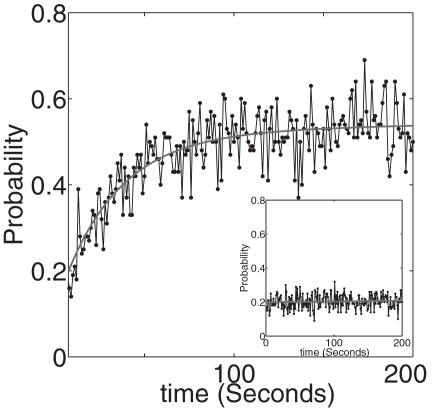
For a synapse that is functionally associated with an astrocyte, the initial neurotransmitter release triggers intracellular Ca^2+^ elevation and a consequential glutamate release from the astrocyte. Due to the positive feedback from the astrocyte, the dynamical increase in release probability (total DP≈3) until it saturates at P≈5 is shown. When the feedback from the astrocyte is absent the release probability fluctuates around P≈0.2 (see inset).

**Figure 6:**
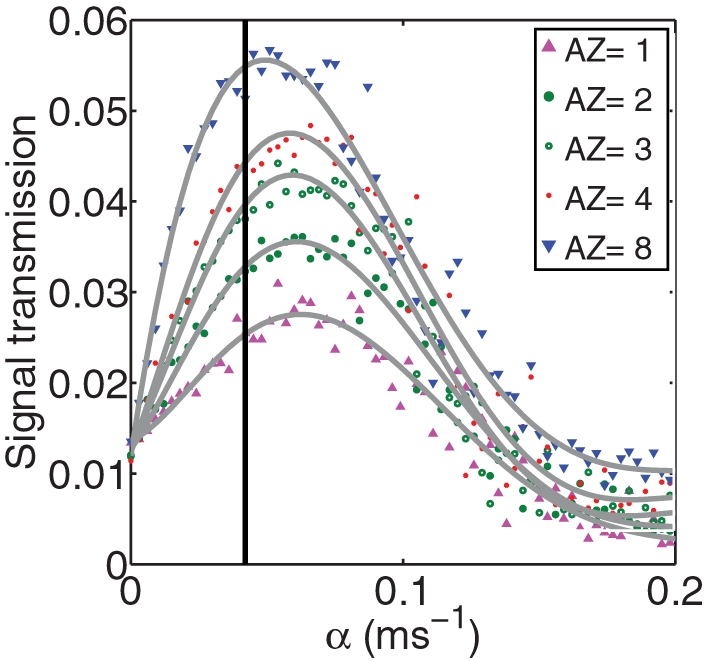
Synaptic transmission (not normalized here, but at a fixed presynaptic spiking frequency of 5 Hz) versus astrocytic feedback. The baseline transmission probability is fixed P≈0.18, while the number of active zones varies. The vertical line denotes the value of α that yields the experimentally measured transmission probability of the tripartite synapse.

We started with a synaptic transmission probability of P≈0.2 and turned on a 20-Hz stimulation. The Bertram model for vesicle release requires a domain presynaptic Ca^2+^ concentration of 300 µM (See Figure 4, top panel) in order to achieve P≈0.2. This led to a rise in single spike release probability (see Figure 5) as the synaptic release leads to calcium buildup in the astrocyte and subsequently to an increase in the presynaptic calcium concentration (see Figure 4, center panel). The increase did not go on indefinitely as the astrocytic response saturates and becomes insensitive to additional glutamate binding. We fixed the value of the astrocytic feedback parameter α governing the presynaptic calcium increase so as to generate the experimentally observed increase of synaptic transmission probability of DP≈0.3 [20]. In addition we varied this feedback parameter (see Figure 6) and plotted the resulting signal transmission level attained after the system had reached a statistical steady-state. Since pre-synaptic calcium is directly modulated by *α*, we obtained essentially the same behavior already shown in Figure 2.

We verified that the increase DP is roughly independent of the stimulation frequency or stimulation regularity, as long as the firing rate is large enough to excite the astrocyte into the oscillatory regime. This arises because of the very weak dependence of the frequency of the oscillatory state on the precise IP_3_ level; in fact, the entire range of frequencies is less than a factor of 2. Furthermore, there is a huge time scale difference between the astrocytic Ca^2+^-dynamics and its presynaptic consequences, and the neuronal spike trains. Thus, the background intracellular presynaptic Ca^2+^ level varies very slowly in comparison to the time-intervals between vesicle-release events and this variation is similar throughout the range of typical hippocampal firing frequencies. Therefore, any moderately active synapse will have its release probability increased by roughly the same amount and it will remain elevated as long as the synapse remains in use.

### The Steady-State Release Probability Is Close to Its Optimal Value

A common variation of synaptic design involves the number of active zones. We extended our calculations to study the astrocytic potentiation of synapses that have the same baseline fidelity (P≈0.18) but have a different number of active zones. The results are plotted in Figure 6. All of these graphs exhibit the expected maximum as a function of the feedback parameter. As the number of active zones increases, the improvement in transmission becomes more pronounced. The vertical line denotes the specific value of α that provides the best experimental fit to data taken from an experiment done on one specific synapse [20] (assumed to contain two active zones). Note that for all these variable synapses, the attained release probability is very close to the one that assures maximal information transfer. This suggests that the inherent bidirectional signaling in the tripartite synapse optimizes synaptic transfer of information.

### The Results Are Robust with Respect to Parameter Calibration

**Figure 7:**
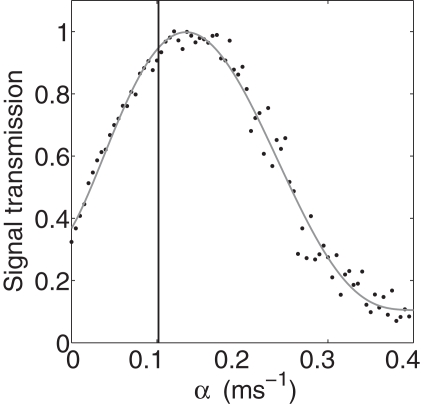
Transmission of information as a function of feedback from the astrocyte under the assumption that the synapse we used for calibration has one active zone for vesicle release. The vertical line denotes the value of the feedback strength (α = 0.101 ms^−1^) that yields the experimentally measured transmission probability of the tripartite synapse.

As discussed in the Methods section, we have chosen our parameters to match experimental data on baseline release probability, its astrocyte-induced increase, and the spontaneous release rate. The match between our model and the data requires an assumption regarding the number of active zones in the measured synapse. All the results presented so far were obtained using two active zones. To test the robustness of our results, we repeated the entire procedure described above under the altered assumption that the experimental data we used to calibrate the model were obtained from a presynaptic terminal with only *one* active zone. This changes the parameters. One active zone requires larger domain presynaptic Ca^2+^ concentration of 430 µM in order to achieve the same baseline transmission probability of P≈0.2. The transmission curves for one active zone and baseline transmission probability of P≈0.2 (analog to Figure 2) for a spike rate of 5 Hz are shown in Figure 7. Again, the curves are almost identical for other frequencies between 5 Hz and 40 Hz (see Figure S2). Whereas the position of the maximum shifts, the conclusion that the value of the feedback extracted from experiment is indeed close to that which optimizes the transmission remains intact. Our results, therefore, are not sensitive to exactly how the model is calibrated with respect to the unknown number of active zones.

### The Enhanced Synaptic Transmission Achieved Due to Astrocyte-Mediation Depends on the Initial Baseline, but the System Remains Optimal

**Figure 8:**
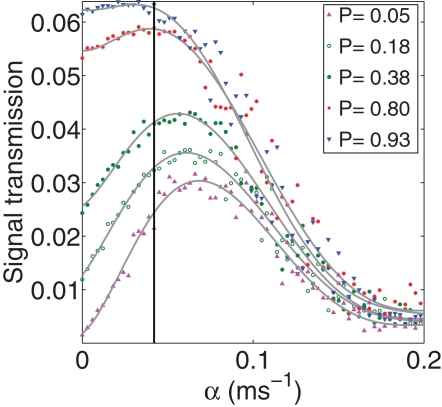
The information transmission (not normalized here, but at a fixed presynaptic spiking frequency of 5 Hz) is shown as a function of the astrocytic feedback at various baseline synaptic transmission probabilities P and a fixed number (2) of active zones. The vertical line denotes the value of α which corresponds to the experimental tripartite synapse.

The notion that the astrocyte will dynamically increase the efficacy of a synapse as it becomes active makes sense for synapses with an initially low probability of release. One might be concerned however that the bidirectional signaling system would act in a deleterious manner for synapses that are intrinsically high-fidelity. We therefore considered the effect of astrocytic feedback on synaptic transmission with a fixed number of (two) active zones, but with a variable baseline (i.e. without astrocyte) transmission probability. While for small baseline transmission probabilities the effect of the astrocytic feedback is a significant improvement of the synaptic transmission probability, this is not the case for large baseline transmission probabilities as seen in Figure 8. Nonetheless, the experimentally measured value of α still corresponds to the optimal value and in particular avoids the region of sharp performance fall-off due to increasing spontaneous release. Of course, it is still the case that the effect of the astrocytes in enhancing synaptic fidelity is most dramatic at low fidelity synapses.

## Discussion

We have developed a modeling framework for the bidirectional interaction between neurons and astrocytes at a synaptic junction. This approach expands upon previous work in two important ways. First, we take into account that the content of synaptic vesicles is variable and can be depressed if the synapse runs short of neurotransmitter resource, and second, that the spontaneous release of vesicles uses up neurotransmitter resource and hence interferes with the transmission of the signal entering the presynaptic terminal as an action potential. This leads to a limit of the enhancement of synaptic transmission through astrocytes and hence predicts that there is an optimal astrocytic enhancement. It is striking that the experimentally observed enhancement lies close to our predicted optimum indicating the possibility that the tripartite synapse **optimizes** synaptic transmission of low-fidelity synapses. [31]. We have further shown that the effect of astrocytes in potentiating higher-fidelity synapses is less dramatic, but the final operating point still retains its near optimality.

In our calculations we do not address the issue of how the feedback parameter α itself might be determined. It thus remains an open question whether longer time feedback mechanisms can automatically find the optimal operating point and hence let the synapse optimize itself. There are a variety of such longer-time processes. Astrocytes drive synaptogenesis through secretion of synaptogenic substances [32], so that in the absence of astrocytes, a variety of synapses are functionally impaired. Astrocytes can secrete additional signals that govern synaptic structure and function dynamically in way that is sensitive to external stimulus over a period of minutes [33]–[35]. They also play an important role in neurons receiving the correct pattern of innervation.

A recent study shows that temporally coincident Ca^2+^ elevations in astrocytes increased transmitter release probability in hippocampal synapses [19], corroborating our modeling assumption. The increase in transmission in this experimental study is due to activation of mGluRs via glutamate release from astrocytes. There are conflicting reports about the modulatory effect of mGluRs on excitatory and inhibitory synaptic transmission. Depression in inhibitory synapses is mediated by activation of group II mGluRs (MCPG sensitive) [36],[37] and activation of group III mGluRs (MAP4 sensitive) leads to depression in excitatory synapses [37],[38], via the inhibition of voltage gated calcium channels. However, our model is based on the activation of MPEP sensitive, group I mGluRs (mGluR1 and mGluR 5), which are coupled to IP_3_ production and which lead to potentiation [18],[19],[24]. We suggest that the seemingly paradoxical outcomes of astrocytic stimulation [18],[37], namely the increase in asynchronous release and the decrease in action potential evoked activity (excitatory and inhibitory) and sensitive to MAP4 and MCPG respectively (Group III and Group II mGluRs) can be consistent. Evoked release is mainly governed by voltage gated calcium channels which can be inhibited by Group III and Group II mGluRs receptors, whereas base level Ca^2+^ which is augmented by Group I signaling regulates asynchronous release. Of course, our model does not consider synapses that only have Group II or Group III receptors.

Another recently published study [39] uses transgenic mice that express G_q_-coupled receptors in astrocytes to show that selective calcium elevations in astrocytes are not sufficient to affect neuronal ionotropic glutamate receptors activity. However, in the same paper they were able to reproduce their earlier finding [18] that IP_3_ uncaging leading to astrocytic calcium elevation causes an increase in spontaneous activity. This enhanced current activity was attributed to enhanced vesicle release due to activation of mGluRs and opening of presynaptic Ca^2+^ stores and therefore remains consistent with our model.

Our calculations demonstrate that the astrocyte-induced enhancement of synaptic transmission depends only weakly on the frequency content of the presynaptic spikes, since the feedback to the neuron reflects integration of neuronal vesicle release over much longer time scales, from seconds to minutes. We also predicted that for a given synaptic baseline transmission probability, synapses with a larger number of active zones are more sensitive to regulation by synaptic astrocytes. While this prediction suggests an advantage for e.g. two versus one active zone, it should not be directly applied to synapses with hundreds of active zones like large calyces and neuromuscular junctions, since their baseline transmission probability is typically very high.

It is generally agreed upon that the number of active zones is 1 or 2 in hippocampal synapses. For a low-fidelity synapse [20] with an intrinsic release probability set for example at P≈0.20, the assumption of 2 active zones implies a presynaptic Ca^2+^ concentration of more than 300 µM during an action potential for the Bertram model [26]. Ca^2+^-influx through voltage-gated Ca^2+^ channels gives rise to microdomain concentration profiles with potentially large concentrations near the channels and large gradients away from the channels. Thus the required 300 µM must be interpreted as a domain Ca^2+^ elevation, which, after termination of the action potential would smooth out rapidly to a much smaller overall concentration by diffusion and binding to buffers. Although such a large value is consistent with values in the literature [40], some recent studies have indicated much smaller values, for example in Calyx of Held [41]. It is important to note, however, that the calyces are large synapses and comprise of 600 or more active zones. A high-fidelity transmission can be achieved in these synapses at much lower presynaptic Ca^2+^ levels since each active zone can independently release vesicles.

Our choice of the small decay-constant for Ca^2+^, released from stores in the presynaptic terminal, (γ in Equation 10 in Methods) reflects the long-lasting synaptic potentiation effect observed experimentally [18],[20]. Although the decay of a micro-domain Ca^2+^ elevation is fast (of the order 100 ms [42]) it has been reported that post-tetanic potentiation, connected presumably with a larger and more global accumulation of presynaptic Ca^2+^, decays only within about a minute [43]. While the decay of *domain* Ca^2+^ is determined by diffusion and buffer kinetics, i.e. local mechanisms, the decay of *global* Ca^2+^ levels is determined by other mechanisms, such as saturated extrusion mechanisms (e.g. Plasma Membrane Calcium ATPase) and/or delayed release from slow buffers if present. Our assumption of a decay time of about one minute is consistent with this overall picture.

Asynchronous release of neurotransmitter by synapses is a poorly understood but a well-established phenomenon. Several parallel explanations have been proposed over the years which include requirement of depolarization along with Ca^2+^ flux for transmitter release [44],[45] making it necessary to postulate voltage fluctuations of the membrane that could lead to release without action potentials. More recent models which do not distinguish between evoked and asynchronous release and thus suggest that Ca^2+^ alone can account for all release [27],[28], could not accurately describe release for low [Ca^2+^] at Calyx of Held synapse. Indeed, our own attempts to directly derive asynchronous release rates for hippocampal synapses with very few active release zones did not succeed, predicting event rates that were much larger than experimental values (with the identical parameters for Ca^2+^ binding we used for induced release). An allosteric model of release was proposed in another attempt to resolve and improve rate predictions [46] but this approach underestimates time-to-peak release rates. A novel study published recently proposes the existence [47] of two distinct Ca^2+^ sensors in a Calyx of Held synapse, one for induced release and another for asynchronous release. The two mechanisms operate independently, with the Ca^2+^ sensor for induced release governing the response during a fast pulse of high calcium concentration and the Ca^2+^ sensor for asynchronous release governing the response during the sustained accumulation of the Ca^2+^ between action potentials. This is consistent with our approach which treats these processes separately. In this study, both mechanisms compete for the same neurotransmitter resource and hence asynchronous release could potentially empty the readily releasable pool during sustained elevations of the Ca^2+^. The two sensor hypothesis is appealing, as it is able to describe a range of dynamics of at the Calyx of Held. We should note, however, that the novel Ca^2+^ sensor for asynchronous release remains unidentified. Furthermore, it is unclear if this hypothesis would remain valid for the much smaller hippocampal synapses with a single active zone.

We conclude the paper by pointing to an interesting parallel with an electrical engineering problem, namely the tuning of a detector in the presence of noise. Let us assume that we want to detect a signal that comes embedded in a noisy background by using a threshold detector. After the detector responds to a signal, it has to recover for a certain time interval before it is ready to detect again. In the absence of noise, the design of the detector is quite simple: the smaller the threshold – the better the result. For small noise the same still holds. But for larger noise, there is an optimal threshold [48]. If the threshold is made smaller than the optimal threshold, the detector spends much time in recovery from noise-events that do not carry a signal – and hence is less able to respond to information carrying signals. If the threshold is larger than the optimal threshold, the detection probability decreases due to poorer sampling of the signal. Here, the detection is equivalent to the release of a vesicle. Feedback from the astrocyte decreases the detection-threshold, as the release of a vesicle is made more likely with increased presynaptic Ca^2+^. Lowering the threshold too much does not help since a) the vesicle release machinery spends much time in recovery and b) neurotransmitter resource is wasted. Conversely, if the threshold is too high, i.e. the presynaptic Ca^2+^ levels too low, the transmission probability of information carrying events is small and the performance of the synapse poor.

## Methods

While several parallel pathways of signaling exist between neurons and astrocytes [15], in this modeling study our goal is to construct a model for the bidirectional signaling pathway between the intracellular calcium dynamics in an astrocytic process associated with a synapse and vesicle release from the presynaptic terminal. Our basic strategy is to combine existing models of the separate pieces and use experimental data to determine unknown coupling terms. For processes such as spontaneous release whose biophysical basis is uncertain, we assume simple phenomenological forms consistent with known biology.

We start with the presynaptic vesicle-release process. Vesicles of neurotransmitter are released from active zones abutting the synaptic cleft. Hippocampal synaptic junctions typically comprise of one or two active zones resulting in zero, one, or very rarely two vesicles released within one action potential. The release of vesicles is controlled by the concentration of presynaptic Ca^2+^, binding to the vesicle release machinery. To describe vesicle release and its dependence on presynaptic Ca^2+^, we use the model by Bertram, Sherman and Stanley [26]. This model assumes that there are 4 binding sites for Ca^2+^, with 4 different binding rates (See Table 1). The binding site j is bound with the rate *k_j_* ^+^ *c_pre_*, where *c_pre_* denotes the presynaptic Ca^2+^ concentration and it dissociates with the rate *k_j_* ^−^.

**Table 1:** Rate constants for the release model by Bertram, Sherman, and Stanley [26].

1/(ms µM)	1/ms
*k*_1_^+^ = 3.75·10^−3^	*k*_1_^−^ = 4.00·10^−4^
*k*_2_^+^ = 2.50·10^−3^	*k*_2_^−^ = 1.00·10^−3^
*k*_3_^+^ = 5.00·10^−4^	*k*_3_^−^ = 0.1
*k*_4_^+^ = 7.50·10^−3^	*k*_4_^−^ = 10.0

Out of the four binding sites for Ca^2+^, two binding sites have slow unbinding rates, *k*_1_^−^ and *k*_2_^−^, (high affinity binding). Arrival of an action potential before Ca^2+^ unbinds from these sites enhances vesicle release probability. This mimics short-term synaptic facilitation consistent with decay times of presynaptic Ca due to a single action potential (100 ms) [49].

As there are only a few active zones, the release process cannot be described by the average release rate, but rather by a stochastic algorithm. Each Ca^2+^-binding site at each active zone can be bound and unbound and transitions occur according to a Markov process. If binding site *j* is unbound at time *t*, the probability *Oj* that it will be bound within the time interval [*t*, *t*+δ*t*] is given by *c*_pre_ *k_j_* ^+^δ*t* for sufficiently small δ*t*. If binding site *j* is bound at time *t*, it will dissociate with the probability *k_j_* ^−^δ*t* within the time interval [*t*, *t*+δ*t*]. A vesicle is released from an active zone at time *t_i_*, when all 4 Ca^2+^-binding sites are bound **and** if the presynaptic membrane is depolarized. Once, a vesicle is released from an active zone, the corresponding vesicle release machinery remains inactivated for about 6.3 ms [29].

In addition to this stimulated release during action potentials, spontaneous release of vesicles can occur even when the presynaptic membrane is not depolarized. The rate of spontaneous release depends on the presynaptic Ca^2+^ concentration [27],[28] in a domain close to the release machinery. Due to the lack of detailed biophysical knowledge of this process, we incorporate spontaneous vesicle release events into our model through a phenomenological release rate
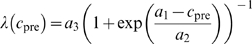
(1)calibrated by recently measured rates of spontaneous postsynaptic current events as modulated by an astrocyte [20]. See Table 2 for parameters. In our computations, the actual spontaneous release events are generated as a Poisson process with the aforementioned rate. Given the times of vesicles release *t_i_*, we determine the amount of released neurotransmitter by using a dynamical model that considers availability and recovery of neurotransmitter resources [50]

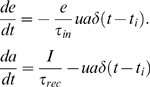
(2)where *I* is *1*−*e*−*a*. At the time of vesicle release *t_i_*, the neurotransmitter content of a vesicle, i.e. *ua*, is released into the synaptic cleft. The amount of neurotransmitter in the vesicle depends on the available recovered resource, *a*, as well the utilization parameter *u*. During a release event the available fraction of resource, *a*, drops by the amount *ua*, but recovers this amount within the recovery time of τ*_rec_*. The fraction of effective neurotransmitter resource in the cleft, *e*, increases upon the release of a vesicle by the same amount, *ua*, but decays subsequently through e.g. pickup by transporters within the inactivation time τ*_in_*. The values of the parameters including *u* are such to best match experimental traces of activity dependent depression (see Table 3) [50]. The amplitude of the postsynaptic current I_post_ upon an action potential depends on the amount of neurotransmitter that was in the released vesicle plus the neurotransmitter that may still be in the cleft, i.e.
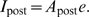
(3)with unit current density *A*_post_. The maximum current in units of *A*_post_ is obtained if all resource was available at the time of vesicle release, i.e. *a* = 1. In general,
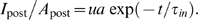
(4)Neurotransmitter released into the cleft also binds to metabotropic glutamate receptors (mGluRs) on the synaptic astrocyte (if present). This in turn can cause release of Ca^2+^ from astrocytic internal stores through generation of the second messenger IP_3_. Such a Ca^2+^ elevation can be local or global. Given the clustered distribution of IP_3_Rs in astrocytes [51] with a typical cluster distance of a few microns and few IP_3_Rs per cluster, we use a simplified stochastic model for the release of Ca^2+^ from store through a single cluster with uniform (averaged) cytosolic Ca^2+^ concentration c_a_ and IP_3_ concentrations *p*. We have modeled this mechanism previously [6] and hence will keep its discussion brief. The astrocytic IP_3_ concentration *p* obeys the balance equation
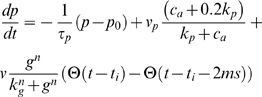
(5)with the linear degradation rate 1/τ*_p_* and a base-level of IP_3_, *p*_0_ [52]. Most importantly, the third term on the right-hand side of Equation 5 is the production rate of IP_3_ upon the release of neurotransmitter from the presynaptic terminal (described by the Heaviside function Θ); *g* denotes the glutamate concentration in the synaptic cleft during the action potential, for which we assume a constant value of 200 mM, consistent with previous analysis [53]. The term has Hill form with a Hill-coefficient *n* and a maximum flux of *v*. The values of these parameters (Ssee Table 4) have been obtained previously by matching frequencies of astrocytic Ca^2+^ concentrations to extracellular glutamate concentrations [54]. When the IP_3_ concentration in the astrocyte is large enough, Ca^2+^ is released from stores thereby causing Ca^2+^ spikes.

**Table 2:** Parameters for asynchronous release (Equation 1).

Parameter	Synapse with 2-AZs	Synapse with 1-AZ
*a*_1_	3022 µM	7181 µM
*a*_2_	261 µM	606 µM
*a*_3_	100 ms^−1^	100 ms^−1^

**Table 3:** Parameters for Tsodyks and Markram model (Equations 2 and 3).

Parameter	Value
τ*_in_*	3 ms
τ*_rec_*	800 ms
*u*	0.45
*A*_post_	1 µA/cm^2^

**Table 4:** Parameters IP_3_ production mediated by neuronal firing (Equation 5).

Parameter	Value
1/τ*_p_*	0.14/s
*p*_0_	0.160 µM
*v_p_*	0.13 µM/s
*k_p_*	1.1 µM
*v*	0.062 µM/s
*k_g_*	0.78 µM
*g*	200 µM
*n*	0.3

For the cytosolic calcium concentration in the astrocyte, we use the relatively simple stochastic version of the Li-Rinzel model [55], i.e.
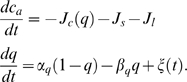
(6)In this model, the astrocytic cytosolic Ca^2+^ concentration c_a_ can change due to three distinct fluxes; Ca^2+^-flux from stores into the cytosol
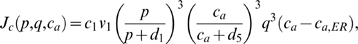
(7a)Ca^2+^-flux through Sarco/Endoplasmic Reticulum Ca^2+^ ATPase (SERCA) from the cytosol to the Endoplasmic Reticulum (ER)
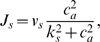
(7b)and leak flux from the ER (high Ca^2+^ concentration) to cytosol (low Ca^2+^ concentration)
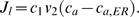
(7c)The form of the flux from ER to cytosol (Equation 7a) has a sigmoidal dependence on IP_3_ and c_a_ and hence reflects the requirement of IP3 and Ca^2+^ as well as positive feedback through calcium-induced calcium release. The variable *q* describes Ca^2+^-induced inhibition when cytosolic Ca^2+^ levels rise too high.

The activation and inactivation rates, *α_q_* and *β_q_*, controlling inhibition through the bottom equation in Equation 6, are given by
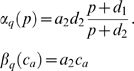
(8)If Ca^2+^ rises too high, the inactivation rate is large, shutting the flux through IP3Rs down. The relatively small number of IP_3_Rs (here 20) generates stochasticity of Ca^2+^ release, which is described (for a systematic derivation, see [55]) in terms of the Gaussian, stochastic force ξ(*t*) with zero mean and correlations according to
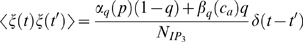
(9)where *N_IP_*_3_ denotes the number of IP3 receptors. All parameters used are listed in Table 5.

**Table 5:** Parameters of the modified Li-Rinzel model for astrocytic Ca^2+^ oscillations (Equations 6–9).

Parameter	Value
*c*_1_	0.185
*v*_1_	6 s^−1^
*v*_2_	0.11 s^−1^
*v*_3_	0.9 µM/s
*k*_s_	0.1 µM
*d*_1_	0.13 µM
*d*_2_	1.049 µM
*d*_3_	0.9434 µM
*d*_5_	0.08234 µM
*a*_2_	0.2 µM^−1^ s^−1^
*N_IP_*_3_	20

In summary, Equations 5–9 describe the astrocytic Ca^2+^ response upon the presynaptic release of a vesicle. In previous work [6] we have compared the time course of the predictions of Equations 5–9 with experimental data [14] and found good qualitative agreement (not fully quantitative, since the fluorescent recordings were not calibrated).

We now turn to the feedback part of the model. Glutamate is released from the astrocytes when its cytosolic Ca^2+^ concentration exceeds a threshold of approximately 200 nM [56],[57] through possibly a vesicular mechanism [58]. Activation of glutamate receptors on the presynaptic terminal due to glutamate released by astrocytes leads to potentiation of synaptic transmission [18]–[20] that lasts for minutes, most likely through release of Ca^2+^ from presynaptic stores. This is a slow process on the time scale of seconds to minutes, which maybe stretched even longer through binding of Ca^2+^ to buffers and/or limited Ca^2+^ extrusion capabilities. In contrast, Ca^2+^ entering the presynaptic terminal during an action potential through voltage-gated Ca^2+^ channels leads typically to an increase of Ca^2+^ on a small domain which decays within about 100 ms after termination of the action potential [49], through diffusion and buffer kinetics. To distinguish between these two types of kinetics, we split the total presynaptic Ca^2+^ concentration *c_pre_* into the local domain Ca^2+^ concentration *c_pre,ap_* entering through voltage gated Ca^2+^ channels and the Ca^2+^ concentration *c_pre,st_* released from the stores and decaying on a time scale of about a minute, i.e.
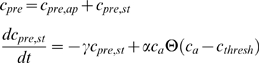
(10)with *c_pre,ap_* fixed and determined by the release probability P of neurotransmitter-filled vesicle within the duration of one action potential. Although we assume that *c_pre,ap_* increases instantaneously when the action potential arrives presynaptically, and switches back to basal level instantly at the end of the action potential, a short-term memory of about 100 ms is taken into account by the small unbinding rates of one of the Ca^2+^ binding sites of the vesicle-release machinery. The second term on the RHS of Equation 10 (source term) describes the linear increase of presynaptic Ca^2+^ if the astrocytic Ca^2+^ concentration *c_a_* exceeds *c_thresh_*. The rate constant γ mimics the time-scale of astrocytic potentiation (see Table 6) [18]. The linearity is a simple assumption in the absence of more detailed information. The actual value of the rate constant α = 0.04/ms for a tripartite synapse has been calibrated for a model with two active zones in order to yield the measured increase in synaptic transmission probabilities ΔP≈0.3 [20] of high-failure synapses after potentiation by the astrocyte. More precisely, given the parameters of the Bertram model, we determined the presynaptic Ca^2+^ concentrations that results in a synaptic transmission probability increase of 0.3 (within the duration of one action potential) and used that to set a.

**Table 6:** Parameters for presynaptic calcium (Equation 10).

Parameter	Value
*c_pre,ap_*	300 µM
*γ*	0.02·10^−3^ ms^−1^
*c_thresh_*	196.4 nM

While our model for the astrocytic Ca^2+^ response to neuronal firing is ***specific*** for the activation of mGluRs and the downstream effect of IP_3_ production and Ca^2+^ release from IP_3_R mediated stores, we implemented a less specific model for the additional astrocyte-induced presynaptic Ca^2+^ to accommodate both NMDA mediated [20],[59] as well as mGluR mediated [18],[19] Ca^2+^ elevation. The only assumption, supported by references, we are making here is that the corresponding kinetics is slow (see Figure 2 of Parpura et al. [60] for Ca^2+^ elevations caused by NMDA receptors).

Our hypothesis of competition for neurotransmitter resource between induced release and asynchronous due to sustained elevation of presynaptic [Ca^2+^] therefore remains valid for both presynaptic activation of NMDA as well as mGluRs, independent of the details of the pathway. Consistent with our assumption, a very recent study [47], reports of competing dynamics of induced and asynchronous release for a single pool of available neurotransmitter and slow, sustained Ca^2+^ being fertile conditions for domination of asynchronous release.

The feedback strength α is the most crucial parameter for this study. It is important to keep in mind that we obtained this particular value for α through calibration with the measured value of transmission probability under the (reasonable) assumption that the presynaptic terminal had 2 active zones. We redid all of the parameter estimation with the alternative assumption that the synapse in question had only one active zone. This changes the parameters (listed in Table 2). As discussed in the text, all of our conclusions remain valid even with this change. A flowchart of the computational model used is provided in supplementary information section (see Figure S1).

## Supporting Information

Figure S1 A flowchart of the computational model used.
(1.37 MB EPS)

Figure S2 The signal transmission (not normalized here) for a synapse with two active zones is shown as a function of the astrocytic feedback for various presynaptic spiking frequencies. The vertical line denotes the value of α which corresponds to the experimental tripartite synapse.
(0.48 MB EPS)
