# Correction: Astrocytes Optimize the Synaptic Transmission of Information

**DOI:** 10.1371/annotation/ec07378d-fd1b-45b8-bfed-1a20c9fd5d26

**Published:** 2008-06-23

**Authors:** Suhita Nadkarni, Peter Jung, Herbert Levine

In the Methods section, Equation 2 is incorrect. Please see the correct equation here:


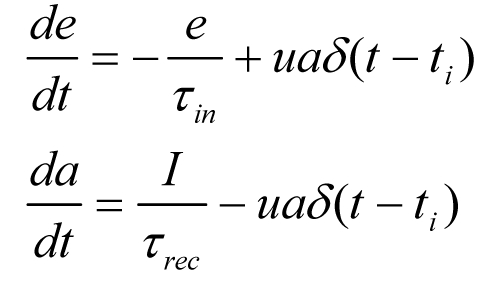
(2)

